# In Vitro Anti-Oxidant and Anti-Microbial Potentiality Investigation of Different Fractions of *Caryota urens* Leaves

**DOI:** 10.3390/biomedicines4030017

**Published:** 2016-07-27

**Authors:** Shofiul Azam, Md. Kayes Mahmud, Md. Hamza Naquib, Saad Mosharraf Hossain, Mohammad Nazmul Alam, Md. Josim Uddin, Irfan Sajid, Muhammad Sazzad Hossain, Md. Salimul Karim, Md. Ali Hasan

**Affiliations:** 1 Department of Pharmacy, International Islamic University Chittagong, Chittagong-4203, Bangladesh; nazmul_pharmacy@yahoo.com (M.N.A.); josim_84@yahoo.com (M.J.U.); 2 Department of Pharmaceutical Sciences, North South University, Dhaka-1229, Bangladesh; kayes.mahmud@northsouth.edu (M.K.M.); hamzah.naquib@gmail.com (M.H.N.); flikhtaan0353@yahoo.com (S.M.H.); irfans011@gmail.com (I.S.); 3 Department of Pharmacy, University of Science and Technology (USTC), Chittagong-4203, Bangladesh; sazzad.ph47@gmail.com; 4 Department of Pharmacy, Northern University Bangladesh, Dhaka-1213, Bangladesh; salimul_k@yahoo.com (M.S.K.); mdhasan97@yahoo.com (M.A.H.)

**Keywords:** anti-microbial, Mueller-Hinton broth, *B. cereus*, *E. coli*, anti-oxidant

## Abstract

Background: *Caryota urens* is a member of the Arecaceae family and a common plant in the Southeast Asian region. This plant has been reported as an anti-microbial agent in recent years. Thus, we aimed to find out the MIC (minimum inhibitory concentration) against different pathogenic microorganism. Methods: The leaves of *C. urens* were extracted and fractioned using different reagents (chloroform, *n*-hexane and carbon tetrachloride). Disc diffusion method was implemented for the assessment of in vitro anti-microbial potency (500 and 250 µg/disc). Result: The entire fraction showed good effect (with the zone of inhibition 19–25 mm) against both gram positive (*Bacillus subtilis*, *Bacillus megaterium*, *Bacillus cereus*, *Sarina lutea*) and gram negative (*Vibrio mimicus*, *Shigella boydii*, *Escherichia coli*, *Pseudomonas aeruginosa*) bacterial pathogens and fungal strains (*Aspergillus niger*, *Saccharomyces cerevisiae*). The plants also possess effective free radical scavenging potency with an IC_50_ of 130.32 µg/mL. Conclusion: This finding reflects a link between the presence of anti-oxidative material and a substantial anti-microbial activity, and substantiates all previous claims against *C. urens*.

## 1. Introduction

In the developing world, medicinal plants make an enormous contribution to the potential source of primary health care necessities. As stated by the World Health Organization (WHO), for medical care treatment, about 80% of populations of the world indiscriminately trust in traditional medicines as their first line therapy. In the developing countries, 3.5 billion people essentially depend on medicinal plants and herbal medicines around them for various medical conditions [1]. In addition, Ayurveda, Unani, and Siddha also provide complementary and alternative medicines to more than 70% of the rural populations [2], to whom the medicinal plants are acclaimed and obvious folk medicines [3,4] because of the assertion of the traditional therapists make that their medicines are not costly and more effective than modern medicines [5]. The folkloristic concepts of medicinal plants play important pharmacological roles in the treatment of diverse medical conditions. The pharmacological actions of medicinal plants are due to the presence of a number of bioactive compounds of which the most significant are alkaloids, flavonoids, tannins, and phenolic compounds [6].

The use of medicinal plants and products is increasing progressively to treat acute and chronic as well as infectious diseases. Infectious diseases have become the most perilous health issues throughout the world [7]. The emergence and re-emergence of pathogenic bacteria, especially multidrug resistant (MDR) bacteria, are a predominant threat to the antimicrobial therapy [8] and considered as a leading cause of drug ineffectiveness and treatment failure [9]. Therefore, these have been disclosed as major cause of morbidity and mortality rates of the population [7]. In recent years, antibiotic resistance is a growing global problem, which necessitates the search for newer, safer, potential and effective antimicrobial agents [10], as the therapeutic choices for notorious MDR pathogens have become extremely limited [11].

Among the potential sources, plants are preferred because they have fewer or no side effects compared with chemical or synthetic substances [12]. The search for natural antimicrobials is a continuous process and their demand is increasing day by day [13,14,15]. Many scientists have already reported very promising findings of antimicrobial activities of extracts of different plants against various microorganisms consisting of pathogenic gram-positive and gram-negative bacteria as well as pathogenic fungi [16,17,18]. It is stated that effective plant extracts can combat human pathogenic bacteria without toxic side effects and environmental hazards, and have other advantages like being cheaper to produce, readily available and easily biodegradable [18]. Pharmacological industries have produced a number of new antibiotics [19,20], but still there is a great need for newer antibiotic therapies to fight the MDR pathogens and minimize the threat.

*Caryota urens* has been studied in recent years and it has been found to have very potent anti-oxidant and anti-microbial activity [21,22,23]. Hence, the present study aimed to scrutinize the antimicrobial activities of tropical *Caryota urens* L. leaves extract against some pathogenic microbes and additionally to perform an antioxidant activity test on the extract.

## 2. Materials and Methods

### 2.1. Collection, Identification and Preparation of Materials

The fresh raw samples of *Caryota urens* were collected from Mirpur Beribadh, Dhaka, Bangladesh in February 2014 and forwarded for identification at Bangladesh National Herbarium, Mirpur, Dhaka, Bangladesh; the Accession number: DACB-39528 was assigned by the authority for *C. urens*. The materials were then delivered to the microbiology Lab of Department of Pharmaceutical Sciences at North South University (Dhaka, Bangladesh).

The aerial parts of the collected materials were washed properly. The leaves were separated and cut into small portions followed by air-drying covered by shade for days. After proper drying, coarse powder of the leaves was prepared using a grinding machine for extract isolation.

### 2.2. Extraction and Fractionation

The sample extract was prepared from 250 g powdered leaves were soaked into 1000 mL of 95% methanol that preserved for 1 week with proper sealing and the mixture was shaken intermittently. Filtration of the mixture was done by cotton filter followed by Whatman No.1 filter paper and the filtrate was subjected to evaporation by rotary evaporator at 50 °C resulting in concentrated crude extract. This extract was then processed for fractionation using *n*-hexane, chloroform and carbon tetra chloride thereafter these fractions were preserved until later use. Yield value of these was also recorded.

### 2.3. Test Microorganisms

For this experiment, the microorganisms were obtained as pure cultures for gram positive (*Bacillus subtilis*, *Bacillus megaterium*, *Bacillus cereus*, *Sarina lutea*) and gram negative (*Vibrio mimicus*, *Shigella boydii*, *Escherichia coli*, *Pseudomonas aeruginosa*) bacterial pathogens and fungal strains (*Aspergillus niger*, *Saccharomyces cerevisiae*) from the Department of Pharmaceutical Sciences, North South University (Dhaka, Bangladesh).

### 2.4. Preparation of Inoculum

Pure cultures of the microorganisms were sustained in nutrient agar media at 4 °C. Subcultures were prepared to be used throughout the experiment by transferring a loopful of cells from the pure cultures to the autoclaved test tubes containing the growth medium: Mueller-Hinton Broth (MHB) for bacteria and Sabouraud Dextrose Broth (SDB) for fungi. After inoculating, the test tubes were incubated at 37 °C and 25 °C respectively for 24 h. These cultures were further diluted with fresh MHB and SDB media to achieve optical densities corresponding to 2.0 × 10^6^ CFU/mL for bacterial pathogens and 2.0 × 10^5^ spore/mL for fungal strains. Aseptic conditions were maintained during the procedure.

### 2.5. Antimicrobial Susceptibility Test

In vitro susceptibility test against the fractionated extracts were performed using the disk diffusion method. In brief, the 0.1% bacterial and fungal suspensions were swabbed uniformly over the surface of the solid Mueller-Hinton agar media using sterile cotton swab under aseptic condition. After complete drying and absorbing of the inoculum, 6 mm sterile filter paper discs were impregnated with the crude extracts at the concentration of 250 and 500 µg/disc and applied to the surface of the inoculated plates allowing to be diffused for 5 min. followed by 24 h incubation. Finally, inhibition zones formed around the discs were measured using a calibrated scale within millimeter. Kanamycin antibiotic disc (30 µg/disc) was used as the standard for both antibacterial and antifungal screening. All the tests for antimicrobial activity were carried out in triplicate.

### 2.6. Minimum Inhibitory Concentration (MIC) Determination Test

The crude extracts were subjected to serial dilution technique in sterile test tubes to prepare various concentrations, for the determination of MIC, from 2 to 512 µg/mL by redoubling rule. The various diluted extracts and sterile nutrient broth medium were added in individual test tubes by equal volume. Afterwards, 10 µL of standardized test organism from the suspension, which requires to be adjusted with 0.5 McFarland standard [20,21], containing 10^7^ to 10^8^ cfu/mL was also mixed well and incubated for 24 h. Three control test tubes comprising as the nutrient medium only (Cm), the medium and sample (Cs) as well as the medium and inoculums (Ci) were carried out to perform the control test. After overnight incubation, the growth of the test organisms was observed to determine the MIC.

### 2.7. Antioxidant Activity Determination by DPPH (2,2-Diphenyl-1-Picrylhydrazyl) Radical Scavenging Assay

DPPH scavenging assay were performed on the previously diluted extracts of *n*-hexane fraction of *C. urens* leaves and the standard was controlled using the same dilutions of ascorbic acid [21].

Percent scavenging of the DPPH free radical was measured using the following equation

(1) $$\%scavenging=\;\frac{Absorbance\;of\;control-Absorbance\;of\;test\;sample}{Absorbance\;of\;control}\;\times 100$$

### 2.8. Statistical Analysis

Analysis was expressed as mean ± SD from three corresponding evaluations. The statistical analysis was performed and the graphical presentations were evaluated using Microsoft Excel 2010 (Microsoft Corporation, Roselle, IL, USA). A p value of less than 0.05 was considered as eloquent. In DPPH scavenging assay, student’s *t* test was used to find the significance of standard and sample for IC_50_.

## 3. Results

A total of eight bacterial and two fungal species were examined for antibacterial and antifungal screening respectively with carbon tetra chloride (250 µg/disc), chloroform (500 µg/disc) and *n*-hexane (500 µg/disc) fractions of extract. Almost all the fractions were effective against bacterial and fungal pathogens despite the fact that carbon tetra chloride extract did not show any significant result against *E. coli*. The findings are summarized in Table 1.

Carbon tetrachloride extract showed significant results against *B. megaterium* with 23.33 ± 2.055 mm zone of inhibition while kanamycin showed 26.33 ± 1.247 mm. On the other hand, the remarkable result of 22.00 ± 1.633 mm inhibited zone diameter for chloroform extract was found against *S. lutea* which is similar to the standard. Chloroform extract showed good activity against gram negative bacteria and at the same time the *n*-hexane extract showed more noticeable antimicrobial effects with 22.6 mm zone, on average, against all the bacterial and fungal strains experimented where 24 mm was the maximum zone of inhibition against gram positive *B. subtilis* and *B. cereus* and 21 mm was the minimum against gram negative *S. boydii*, *E. coli* and *P. aeruginosa*. Moreover, the diameters of the zones were close to the standard kanamycin except *E. coli*.

Among the fractions, the *n*-hexane methanolic extract of the crude *C. urens* leaves was further scrutinized for the MIC against all the above-mentioned organisms (Table 2). The minimum concentration of the extract that did not harbor the organism was specified as the MIC value. The noteworthy antibacterial activity was observed against *B. subtilis*, *S. lutea* and *V. mimicus* at MIC 64 (µg/mL) followed by MIC 128 (µg/mL) against *B. megaterium*, *B. cereus* and *E. coli*. In contrast, antifungal activity was observed against *A. niger* and *S. cerevisiae* at MIC 128 (µg/mL).

**Figure 1:**
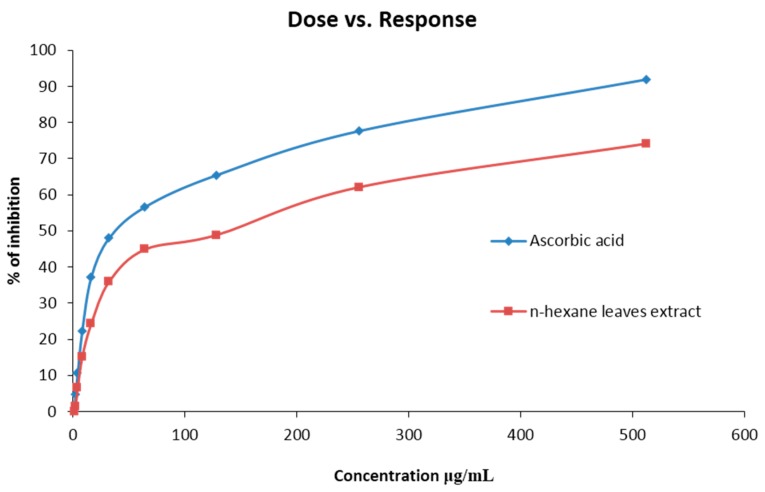
DPPH radical scavenging activity of crude methanol extract of *Caryota urens* and Ascorbic Acid (Standard).

**Figure 2:**
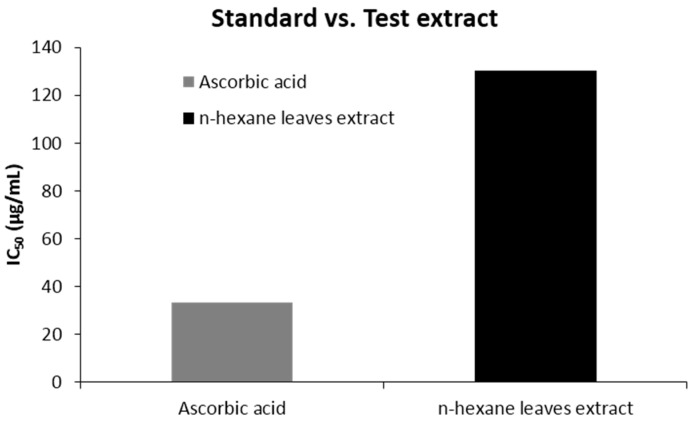
IC_50_ (μg/mL) values of *n*-hexane leave extract of *Caryota urens* and ascorbic acid for DPPH radical scavenging activity.

The percent scavenged DPPH free radical for the extract was found to be less than the standard ascorbic acid. At a concentration of 512 (μg/mL), the scavenging activity was found 92.45% for ascorbic acid and 74.12% for the extract, which is in a ratio of 1:0.8 (Figure 1). The IC_50_ value was 130.32 (µg/mL) for *n*-hexane extracts. The scavenging activity found at different concentrations is summarized in Table 3 and Figure 1 & Figure 2.

## 4. Discussion

*Caryota urens* is an Asian species, used traditionally in the treatment of gastric ulcer, migraine headaches, snakebite poisoning and also rheumatic swellings by preparing porridge from the flowers [24]. Additionally, in Ayurveda, *C. urens* is suggested to treat seminal weakness and urinary disorders [24]. In the present study, *C. urens* leaves were principally investigated for the presence of antimicrobials activity along with antioxidant activity. Emphasize given was more on antibacterial properties, since the bacterial infections are observed to be more prominent among the microbial infections, wherein bacterial infections account for 90% [25].

The disk diffusion method is the oldest and most widely used antimicrobial susceptibility testing method because of its suitability for testing majority of the microbial pathogens [26] and this method is also very important and effective for qualitative antibacterial screening of crude drugs [27]. In addition, the dilution method is important for quantitative screening where the minimal inhibitory concentrations are determined [27]. The MICs are referred to as the gold standard for determining the susceptibility of organisms to antimicrobials [28]. A lower MIC value indicates the necessity of less amount of drug to inhibit the growth of organisms.

However, in our study, although almost all the extracts showed noticeable activity in agar diffusion test against the examined pathogens, more expressive activity was revealed for *n*-hexane methanolic extract. The most effective antibacterial activity was found against two gram positives, *B. subtilis*, *S. lutae* and one gram negative, *V. mimicus* at 64 (μg/mL). Moreover, two other gram negatives *P. aeruginosa* and *S. boydii* required MIC value of 256 (μg/mL) concluding that the *n*-hexane extract was more active against gram-positive bacteria. The differences in the result can be described by studying the results from previous findings where most of the plant extracts showed greater activity against gram-positive bacteria [11,29,30]. The difference in the nature of the cell wall of the gram positive and gram-negative bacteria could be one of the practicable reasons for this kind of observation as the narrow porins in the gram-negative bacterial cell wall inhibit the penetration of the molecule inside the bacterial cell, which is not associated with gram-positive bacteria [31]. The difference in the efflux pump of the gram negative could also be another reason for this difference [32]. Recent studies on antimicrobial effect of *C. urens* had shown that the methanol extract of the leaves is also effective against some other bacteria such as *S. aureus*, *V. cholera*, *S. typhi* and *S. flexneri* [33].

Along with the antibacterial activity, the antifungal activity tested in the same manner also gave a moderate outcome having competent zone of inhibition. Although pathogenic fungi are considered as the leading infectious agents in phytopathology [34], studies on clinical investigation of *A. niger* and *S. cerevisiae* demonstrated that these could be the reason for significant morbidity and mortality [35,36]. In that reflection, our findings revealed justifiable antifungal activity against those fungi having a MIC value of 128 (μg/mL) for *n*-hexane extract of *C. urens* leaves.

Oxidative stress is the phenomena generated due to the presence of excess amount of free radicals inside the body, which is the consequence of the imbalance between the formation and neutralization of reactive oxygen species [37]. To prevent the induction of variety of chronic disorders, namely cancer, CVS (Cardio Vascular System) disorders, CNS (Central Nervous System) disorders, pulmonary disorders, rheumatoid arthritis, ocular and fetal disorders due to oxidative stress, the excess radicals should be regulated properly by providing antioxidants externally [38]. Studies recommend that the natural phytocompounds are an excellent source of antioxidant agent [38]. The subsequent investigation of our *n*-hexane leave extracts for oxygen scavenging activity compared with the ascorbic acid standard showed a dose dependent action: the more the concentration, the more the percent scavenged. The IC_50_ value for the extract was almost 4 times that of the standard, which might indicate a moderate level of antioxidant activity. From the previous studies, it is suggested that, the antioxidant activity of plant extract differs based on the fraction of the extracts used and the scavenging assay techniques [22,39]. According to Md. Sahab Uddin et al., 2015, the *n*-hexane extract of this leave has low level of antioxidant activity compared to other fractions, which is slightly inconsistent with our finding [40]. In another study, it was demonstrated that *C. urens* leaves contain ample amount of phenolics and flavonoids [22,41] and it is already advocated that plants containing phenolics and flavonoids could have good in vitro antioxidant activity [42] wherein supporting this statement, studies revealed that the strong antioxidant potential of *C. urens* leaves was confirmed after finding the chemical constituents by GC-MS [34] and with significantly high radical scavenging activity [22].

Previous reports suggest that phenolic and flavonoid compounds (gallic acid, caffeic acid, *p*-coumaric acid, quercetin, rutin and catechin) in plants possess strong antioxidant activity that might contribute to antimicrobial potential [33,43]. The disc diffusion method was employed for the determination of antimicrobial activity of plant extracts against different human pathogens. The zone of inhibition of the plant extract was found to be in the range from 17 to 25 mm (Table 1) against the entire tested microorganisms. Different fractional extracts from leaves of *C. urens* showed maximum activity of about 25.87 mm against *S. boydii* (chloroform extract) and against *E. coli* extract showed 25.63 mm of zone of inhibition. Plant based antibiotic drugs have enormous therapeutic potential and have been proven effective in the treatment of infectious diseases with less or no side effects which are often associated with synthetic antibiotics. The terpene alcohols damage the cell membranes of *E. coli*, *B. subtilis* and *S. lutae*, resulting in leakage of potassium ions from cells, which cause death of the organism. Recent reports suggest that the sesquiterpene alcohol like farnesol has been confirmed to reduce the growth of *S. aureus* [44,45]. From previous study, it has been identified that the major antimicrobial compounds such as 10-undecenoic acid, caffeine and 2E,6E-farnesol are present in the *C. urens* [33], which may have many pharmacological activities and the results from our investigation also support that claim. Moreover, it is also reported that compounds in lower quantities might be involved in some type of synergism with the active compound which might be the reason behind the high activity of antioxidant and antimicrobial in leaves of *C. urens*.

Unlike antioxidant test studies, antimicrobial activity of *C. urens* leaves was not previously strongly demonstrated. Nevertheless, in one study, it was suggested that the antimicrobial activity evinced by the leaf extract is the reason for the existence of antioxidants [33]. From our study, we can undoubtedly and certainly ensure the presence of pharmacologically active compounds in *C. urens* leaves having antimicrobial as well as antioxidant properties. Our findings revealed a potent in vitro activity of the *C. urens* leaves, which may not be consistent with the activity if searching for in vivo results, as there remains a chance of the active compounds being degraded or metabolized inside the living system. In the future, further epidemiological and clinical studies will be important to interpret the efficacy of use of *C. urens* leaves extracts.

## 5. Conclusions

In our study, we assessed primarily the antimicrobial and then antioxidant activity of *Caryota urens* plant collected from Dhaka and a potent antimicrobial activity was observed in vitro stipulating the fact that *C. urens* could be a good source of antimicrobial alternatives. Even though our experiment significantly described the potential antimicrobial activity against few microbes, further detailed investigation, such as antiviral action, antimycobacterial action, antiparasitic action, toxicity, and most importantly in vivo efficacy, should be executed to establish the antimicrobial action properties and provide a tailor made alternative source to combat infectious agents.

## Figures and Tables

**Table 1:** Inhibition zone of *C. urens* leaves extracts against microorganisms.

Diameter of Zone of Inhibition (mm)				
Test Organisms	Carbon Tetra Chloride (250 µg/disc)	Chloroform (500 µg/disc)	*n*-Hexane Extract (500 µg/disc)	Kanamycin (30 µg/disc)
Gram Positive Bacteria				
*Bacillus subtilis*	18.67 ± 1.247	21.00 ± 1.414	24.67 ± 1.247	27.67 ± 1.700
*Bacillus megaterium*	23.33 ± 2.055	17.67 ± 2.494	21.00 ± 3.266	26.33 ± 1.247
*Bacillus cereus*	19.33 ± 0.943	20.33 ± 0.471	24.00 ± 3.266	27.33 ± 2.494
*Sarina lutea*	21.00 ± 0.816	22.00 ± 1.633	21.00 ± 1.633	24.67 ± 1.247
Gram Negative Bacteria				
*Vibrio mimicus*	17.00 ± 1.633	23.00 ± 2.449	22.33 ± 1.700	28.67 ± 1.247
*Shigella boydii*	17.33 ± 2.494	25.83 ± 0.850	21.17 ± 1.434	28.17 ± 1.027
*Escherichia coli*	Not significant	25.67 ± 1.247	20.67 ± 0.943	34.33 ± 3.300
*Pseudomonas aeruginosa*	20.33 ± 2.055	22.67 ± 2.055	21.33 ± 1.247	26.00 ± 2.944
Fungi				
*Aspergillus niger*	18.67 ± 2.055	26.67 ± 1.247	21.67 ± 1.247	23.00 ± 0.816
*Saccharomyces cerevisiae*	22.33 ± 0.943	23.33 ± 0.471	24.00 ± 0.816	25.33 ± 0.471

**Table 2:** Minimum Inhibitory Concentration (MIC) (µg/mL) of fraction of *n*-hexane methanolic extract of *Caryota urens* L. leaves.

Sample	Concentrations of *n*-Hexane Extracts (µg/mL)	Observation of the Growth Results of the Microorganisms at Different Concentration									
		Bs	Bm	Bc	Sl	Sb	Pa	Ec	Vm	An	Sc
1	512	NG	NG	NG	NG	NG	NG	NG	NG	NG	NG
2	256	NG	NG	NG	NG	NG	NG	NG	NG	NG	NG
3	128	NG	NG	NG	NG	SG	SG	NG	NG	NG	NG
4	64	NG	SG	SG	NG	SG	SG	SG	NG	SG	SG
5	32	SG	G	G	SG	G	G	G	SG	G	G
6	16	G	G	G	G	G	G	G	G	G	G
7	8	G	G	G	G	G	G	G	G	G	G
8	4	G	G	G	G	G	G	G	G	G	G
9	2	G	G	G	G	G	G	G	G	G	G
Cs	512	NG	NG	NG	NG	NG	NG	NG	NG	NG	NG
Ci	0	G	G	G	G	G	G	G	G	G	G
Cm	0	NG	NG	NG	NG	NG	NG	NG	NG	NG	NG
MIC (µg/mL) determined from *n*-hexane extract		64	128	128	64	256	256	128	64	128	128

Bs: *Bacillus subtilis*; Bm: *Bacillus megaterium*; Bc: *Bacillus cereus*; Sl: *Sarina lutae*; Sb: *Shigella boydii*; Pa: *Pseudomonas aeruginosa*; Ec: *Escherichia coli*; Cm: *Vibrio mimicus*; An: *Aspergillus niger*; Sc: *Saccharomyces cerevisiae*; SG: Slight growth; NG: No growth; G: Growth.

**Table 3:** Percent scavenged DPPH free radical at various concentrations for Ascorbic acid and *n*-hexane leave extract of *C. urens*.

Sample	Concentration (µg/mL)	% of Inhibition	IC_50_ Value (µg/mL)
Ascorbic acid	1	0.95	33.18
	2	4.55	
	4	10.36	
	8	22.45	
	16	37.15	
	32	48.22	
	64	57.85	
	128	65.56	
	265	78.22	
	512	92.45	
*n*-Hexane leave extract of *C. urens*	1	0.35	130.32
	2	1.23	
	4	6.45	
	8	15.24	
	16	25.36	
	32	36.47	
	64	45.25	
	128	49.11	
	265	62.45	
	512	74.12	
